# Notes on the Anatomy of the Dolphin’s Brain

**Published:** 1886-04

**Authors:** 


### EDITORIAL DEPARTMENT.

### NOTES ON THE ANATOMY OF THE DOLPHIN'S BRAIN.

Through the kind mediation of Mr. Eugene G. Blackford, of Fulton Market, New York City, Dr. E. C. Spitzka was enabled to obtain a large dolphin from the shores of North Carolina. The animal in question, which weighed 286 pounds, and was about eight feet long, belonged to the species known as the bottle-nosed dolphin (*Tursiops tursio*), and was a male. The brain weighed 2 pounds 12 ounces, and 5 1-2 drachms, apothecaries weight, that is a little over fourty-four and a half ounces, which is about the average weight of the human brain. Dr. Spitzka found the entire length of the spinal cord, measured from above the first cervical nerve roots to the end of the *conus terminalis*, to be 57 centimeters (22 1-2 inches), and relatively to the length of the animal, the shortest in the animal kingdom, reaching only to the middle of the dorsal fin. It was remarked that there was a singular disproportion in the length of the spinal segments; those of the cervical and lumbar region being very short, and those of the dorsal region very long, exaggerating the disproportion common among the mammalia.

| ALTITUDE. | WIDTH OF SEGMENT. | LENGTH OF SEGMENT. | SPECIMEN NUMBER |
|---|---|---|---|
| 4th Cervical Nerve. | 12 Millimeters. | 9 Millimeters. | —— |
| 8th " " | 11 1-2 " | 13 1-2 " | xxxi. |
| Upper Dorsal. | 10 1-2 " | 27 " | xxiv. |
| Middle Dorsal. | 9 1-2 10 " | 27 1-2 " | xxii. |
| Upper Lumbar. | 10 1-2 " | 13 " | xvi. |
| Middle Lumbar. | 10 " | 8 1-2 " | xii. |
| Lower Lumbar. | 9 " | 7 " | viii. |
| Upper Conus. | 6 1-2 " | 4 " | ii. |

Below the lower lumbar level, the total length of the conus terminalis is 36 millimeters, it tapers gradually, and is rather blunt at the end. In man and in the mammalia generally, where the posterior extremities are well developed, the nerve roots from the lumbo-sacral enlargement of the cord are very much larger than those of the cervico-brachial enlargement; in the porpoise as in the seals, they are much smaller; indeed they are in the porpoise almost filamentous as compared with the powerful strands found in land animals, and the posterior nerve roots are much less voluminous than the anterior, with which fact the atrophy of the post cornu, particularly in its gelatinous portion seems to be in harmony.

As if to show beyond peradventure that the porpoise—for the dolphin, like the whale and grampus, is a member of the same family—is an animal depending on an exquisite space sense as a guide in its intricate movements, the auditory nerves were found to be of unusual dimensions; their transection resembled squares, with rounded angles, and they measured exactly seven millimeters in both short diameters. In other words, both auditory nerves together measured in the square area of their trans-section about 100 square millimeters, while the lower lumbar cord of the same animal measured only 85 square millimeters!

Dr. Spitzka states that new dissections will be necessary to demonstrate whether the demarcation between the oblongata and cord be as distinct in the porpoise as in the mammalia usually studied; certainly the transition is extremely gradual. The fourth ventricle is unusually long, measuring 55 millimeters in length. The *eminentia facialis* shows with uncommon distinctness (as the abducens nucleus does not rise up to disturb it); it is a straight elevation, running in the sagittal plane, and rising convex in the middle. It is similar as are also other venticular features to that of the horse, only that in the latter animal, the facial eminence is a little concave outwards. The length of each eminence, cephalo-caudad, is 10 millimeters; the width $2\frac{5}{6}$ millimeters, and the distance between the two $4\frac{3}{4}$ millimeters. In no animal thus far examined does the median sulcus of the ventricle, sink so deeply in the anterior quarter as in the porpoise. An elevation is noted on the antero-lateral boundary of the ventricle, which runs as a distinct columnar fasciculus on the inner side of the post-optic lobes, into the lateral wall of the aqueduct, where it is apparently continued for some distance.

The facial eminences pretty accurately divide the ventricle into an anterior and posterior even half, as the distance from their posterior dive to the obex, is 23 millimeters.

When the enormous size of the cerebral hemispheres is considered, the smallness of the corpus callosum, which has been noticed by earlier observers, appears remarkable. Its length, absolutely is 50 minimums, but straightened out it would measure 7 millimeters more. Its *genu* and *spleniun* each measure 5 millimeters in thickness, but its *corpus* only 2 millimeters. Dr. Spitzka is unable to find an anterior commissure (*precommissura*). There are rudiments of the inner division of the olfactory peduncle.

The discrepancy between the anterior and posterior pairs of the copora quadrigemnia is very great; in fact no animal with physiologically active visual organs shows so great a relative reduction of the anterior pair, except the opossum and the smaller bats; and even these do not show it to the same extent. The post-commissure is enormous, measuring 7 1-2 millimeters, dorso-ventrad and the supra-commissure, to which, as far as we know Wilder first directed attention, is exceedingly well developed also. The miscalled middle commissure shows the usual relations; there is a *distinct fifth ventricle* (pseudocele) and the pellucid septa resemble the human remarkably. The inner geniculate bodies are proportionate in size to the postoptic lobes, but not as convex as in the seal.

In his article on the Pyramid Tract, published in our last number, Dr. Spitzka spoke of a transverse mass behind the pons, about whose relations he was in doubt, and supposed it might be in relation with the auditory nerves. The excellent specimen now obtained, permits him to declare that *it is the trapezium*, which some recent authorities regard as connected with the auditory nerves, and he is thus able to add another to his numerous grounds for asserting that the cetacea have no pyramidal, *i. e.*, voluntary controlling tracts for digital motion, inasmuch as the trapezium extends uninterruptedly across, not being concealed near the middle line, as it is by the pyramid tracts in other animals.

The ventral fissure of the cord, becomes shallow, and ceases at the transition to the oblongata. The olives are rounded eminences, as described and figured by Leuret, and each has a slight latero-cephalic appendix.

Dr. Spitzka's statement comparing the elephant's brain to that of the cetacea, in which it is intimated that, to convert the former's brain into the latter, the frontal lobes should be curtailed and tilted, must be modified, and a definite opinion reserved, until the homologies of the fore-shortened brain of the porpoise are better understood. The most important projection tracts of the cetacea are the auditory and trunk-muscle nerves, and it is in accordance with this fact that we find the auditory nerve and the posterior commissure so very large. There could be no better illustration of the fact that the central nervous system in its morphology is a guage of the entire animal, reflecting its surrounding circumstances, than that of the dolphin. Wundt called the soul the "mirror of the external world," we may surely then designate the brain and spinal cord of the cetacea as the reflection of free ocean life. Here there are no solid rests for the feet, no claws to rend the prey. The gladiator grampus (*orca*) rushes among the other fish, snapping them up and swallowing them in its mad careering race, without any other guide than its accurate guage of distance and skill in trunk motion; porpoises tumble along at the rate of speed of a first-class frigate, and cohabit while so doing; the largest whales raise their huge forms out of the water, race along in herds, with swift and intricate gambols, and do not once come in collision. Obviously the main dependence of these animals in co-ordination is the action of the axial muscles, it is these that Meynert suspected to be under the control of the thalamus, and it is the thalamus and the thalamic tracts which preponderate in the cetacea. This single fact associated with the corelated absence of the pyramids accounts for the following: 1st, The large size of the posterior commissure; 2d, The absence of descending pons fibers; 3d, The large size of the tegmentum; 4th, The prominence and collision of the olives; 5th, The absence of a true ventral fissure for the whole length of the oblongata; 6th, The greater depth of the ventral fissure of the cord as compared with the dorsal fissure, and 7th, The preponderance of tracts in the tegmentum, whose homologues are not easily found in man.

Through the kindness of Professor Wilder, Dr. Spitzka had an opportunity of examining the hind brain of the manatee, as well as its cord. Some of the results are so interesting that we take the opportunity of presenting them with the sanction of both gentlemen, and with the express recognition of the former's right of priority in the elaborate and valuable field of inquiry he has made his own. The brain was in beautiful condition, Professor Wilder having preserved the entire animal and all its organs as a whole, by his method of continuous injection of alcohol.* The beauty of the ventricular cavities can only be appreciated by him, who has seen a brain preserved by this method. The facial eminences are apparently as prominent as, but less distinctly marked than in the porpoise; the entire organization of the pons and oblongata is low. While the pons in the dolphin† compares favorably in cephalo-caudad dimensions with the human, that of the manatee is no longer than that of the hippopotamus, and is shallow throughout. The diameter of the oblongata dorso-ventrad is remarkably even with that of the cord.

There is neither olivary body nor pyramid visible on the ventral face, but the eminence *A*, of Dr. Spitzka's diagram of the hippopotamus is distinctly marked, as it is also in the horse. The boundary between the posterior border of the pons and the oblongata, is scarcely to be identified near the medium line while near the cerebellum (medipedunculus) it is quite as well marked, and proportionately of about the same size as in the hippopotamus. The cerebellum of the manatee shows distinctly the ungulate type, which cannot be said of the cetacea who approximate the proboscidea on the one hand and in general physiognomy—even higher types. Dr. Spitzka states that the brain axis of the manatee presents the least distinct sculpturing among the mammalia. Taking the average development of the domestic mammals—be they carnivores or ungulates as the type; that of the manatee appears as a degradation, and that of the cetacea as a higher development. Is it to be wondered then that the *Rhytina Stelleri*, the Arctic manatee became extinct?

But even in this degraded mammal, a beautiful physiological contrivance is found: The hind brain and spinal cord are actually floated in an intricate plexus of blood vessels. It is true that the spinal cord of the porpoise is provided for in the

---

*Alinjection. †It is shallow behind, but deep in front.

same way, so that Dr. Spitzka was able to remove it without injury, by simply smashing the vertebral arches, but the plexus does not extend so far cephalad, to the disadvantage of brain development. It is perhaps difficult for him who has not the brain and its relations continually before him, to conceive the enthusiasm of him who having discovered in the pyramid atrophy of the porpoise, the reason why it is not a prehensile animal, sees in the defective pons of the similarly shaped but clumsy and less intelligent manatee, the reason why it does not race in mid ocean and tumble in the surf like the swift and intelligent cetacea.

The following series showing the intellectual rank (?) of animals, as guaged by the proportion of the square cord area to the cubic brain area, demonstrates the high position of the porpoise. Man, 50; porpoise, 76; ara parrot, 153; baboon, 177; sea-lion, 198; sun-bear, 217; horse, 294; squirrel, 1555; opossum, 1653; alligator, 3682; iguana, 4611. C.

---

Rabies.—From the Fortschritt d'Medicine we find this disease has suffered a most marked decrease in Bavaria during the last few years. In the year 1875, 485 dogs, 7 horses, 40 cattle, 14 sheep and 6 swine died from the disease. During the last seven years, there have been altogether 370 cases of rabies in dogs, 7 horses, 9 cattle, 7 sheep, and 5 swine, 39 human beings were bitten—fatality not given.

In Vienna, 196 dogs were admitted to the Veterinary Hospital during the year 1884, as either rabid, or suspected. Of these, 178 were living when brought to the school; 134 of the same died of furious rabies, and 27 of the dumb, or still form.

Forty-seven head of cattle were bitten by rabid dogs and died from the disease in one locality in Prussia, in the year 1883. Seventy-four persons were bitten by rabid dogs, in Prussia, in the year 1884-5; four died. Period of incubation 21, 39, 77 and 156 days.

---

Science and the State.—Our readers will find this subject fully discussed in "Mind and Nature," for February, by Dr. R. W. Shufeldt, U. S. A. No impartial thinker on the subject will deny that science exercises a powerful influence in the advancement of all matters pertaining to the welfare and progress of human affairs, nor that good government is an important factor in promoting it. We firmly believe so and would recommend our Legislature to hasten to avail itself of such a valuable guide as science has proved itself, and to do the utmost to aid it. In no way could it do so better than by endorsing the plan submitted by the author of the above-entitled paper and putting it into operation as soon as possible. C.

---

Trichiniasis in Prussia in 1884.—In the Archiv fur Thierheilkunde, we find there was an average of 1 Trichinous in every 1,741 hogs examined in Prussia during the year. The number varied very much in different localities, for which there must be some ascertainable reason—it prevails much more in the hogs of East than in those of West Prussia. In Posen 744 trichinous were found among 154,004 swine: 1 to 193; again at Bromberg of 43,345 swine examined, 161 were trichinous: 1 to 269. In Dusseldorf, only 1 trichinous hog was found among 57,882: at Wiesbaden, 1 to 20,672; at Muiden, 1 to 30,146; at Arnsberg, 1 to 12,362. At the Berlin Abbatoir, 196 trichinous were found in 258,538 hogs, 1 to 1,311.

Trichinæ were found in 41 of 1,724 pieces of American pork at Stettin, but such statistics have no comparative or reliable value whatever.

In the village of Norndorf, having 600 inhabitants, there was an endemic of trichiniasis complicating 86 people—12 died. One hog was the cause of it all. The hog was examined by an examiner, and pronounced "free" from trichinæ, but the result found that he could not be trusted.

In another place, there were 17 cases in human beings, of whom 4 died.

Measels is very prevalent in German hogs.

---

Castration of the Horse.—A number of our readers have asked for the mode of treatment after the ecraseur has been used on the horse. It is conceded by professional castrators who use the ecraseur that 24 hours after the operation, when swelling takes place, the wounds should be kept open by inserting the four fingers, bathing the parts with hot water once or twice a day, keeping the end of the spermatic cord free from attachment to the integuments, and driving the animal daily. The object of the latter is to prevent the parts from swelling. When driven daily or exercised freely, the swelling decreases, the wound heals quickly, and no bad results follow.

---

TUBERCULOSIS IN THE HORSE.—Recuill d. Med. Vet. 1884, Trasbat reports a case of this very rare complication in the horse, which on that account is worthy of consideration in detail. He first mentions having presented to the Société (Central de Med. Vet.,) in April, 1878, the lungs, liver, and organs of a horse that had been killed on account of suspected glanders, but the autopsy revealed phenomena, which differed essentially from the pathological characteristics of glanders, sarcoma, carcinoma, and lymphadenitis, and which he then looked upon as speaking decidedly for the tuberculous nature of the disease. The gentlemen present did not, however, coincide in this conclusion. Lately another case came to hand which he investigated most carefully in connection with his colleague Professor Norcord, and successfully proved the disease to be tuberculosis.

The microscopic examination of the neoplasms revealed by the autopsy, gave unmistakable evidence to the presence of tubercle bacilli in great numbers; the sections having been treated by the differentiating method of Ehrlach.

The chief intra vital phenomena were; an excessive degree of emaciation; augmenting labored respiration without any ascertainable indications of the existence of pneumonia; a short, painful cough, with some nasal discharge, continuous fever with exacerbation evenings; fitful appetite, bronchial rales, polyuria to an excessive degree. He suggests the possible, practical value of the examination of the nasal discharges in such cases.

Autopsy: The spleen was normal in form but weighed two kilos, its entire surface was covered with numerous neoplasms of variable dimensions and color, its pulpa was also invaded by the same; cheesy degeneration of their center failed. A large tumor with caseous degenerated interior situated behind the pancreas. Lungs failed to collapse on removal. Pluræ smooth, with a few circumscribed indurations here and there. Numerous neoplasms distributed through their substances from sub-milliary to the size of a walnut; they all had a spherical form and were not sharply circumscribed in the pulse-tissues, the larger nodes caseously degenerated in center. They developed in the interlobular tissues and caused atrophy and compression of the pulmonary tissue. Respiratory mucosa normal except in bronchials, where there was catarrhal swelling and viscid secretion. Cause unknown.

---

TUBERCULOSIS IN A DOG.—At the meeting of the Société centrale de Med. Vet., 26th February, 1885. Nocord reports a case of Dr. Andrien, in which he found tubercle bacilli in the organs sent to him for examination.

Autopsy: Muscles anæmic, abdominal cavity contained a quantity of clouded fluid; omentum and its lymph glands—latter hypertrophied—the seat of numerous milliary and sub-milliary noduli; dispersed neoplasms in the capsule of the liver and spleen as well as the serosa of the bladder. Considerable senus effusion in thorax; pleura indurated here and there, in such places numerous noduli as above were to be seen. Bronchial lymph glands hypertrophied, on cutting cross section cavity in the body of gland, noduli dispersed through the lungs. Heart, trachea, and larynx, normal.

---

GLANDERS AND PLEURO-PNEUMONIA IN PRUSSIA.

Fifteen hundred and twenty-five horses were killed by the authorities, on account of glanders, during the year 1884–85. The sum of $87,698.24 was paid as remuneration to the owners of the animals. Three thousand and eighty-four cattle were also killed by the authorities on account of the contagious lung plague; six per cent. of these were found to be free from the disease on being slaughtered. The owners received in remuneration the sum of $167,776.02.

